# Authenticity and Relationship Satisfaction: Two Distinct Ways of Directing Power to Self-Esteem

**DOI:** 10.1371/journal.pone.0146050

**Published:** 2015-12-31

**Authors:** Yi Nan Wang

**Affiliations:** School of Psychology, Beijing Normal University, Beijing, 100875, China; University of Vienna, AUSTRIA

## Abstract

Possessing power contributes to high self-esteem, but how power enhances self-esteem is still unknown. As power is associated with both self-oriented goals and social-responsibility goals, we proposed that power predicts self-esteem through two positive personal and interpersonal results: authenticity and relationship satisfaction. Three studies were carried out with a total of 505 Chinese participants, including college students and adults, who completed surveys that assessed personal power, self-esteem, authenticity, relationship satisfaction, communal orientation, and social desirability. Hierarchical multiple regression analyses demonstrated that power, authenticity, and relationship satisfaction each uniquely contributed to self-esteem. More importantly, multiple mediation analysis showed that authenticity and relationship satisfaction both mediated the effects of power on self-esteem, even when controlling for participants’ communal orientation and social desirability. Our findings demonstrate that authenticity and relationship satisfaction represent two key mechanisms by which power is associated with self-esteem.

## Introduction

Power considerations are ubiquitous in daily life [1,2]. Recent studies show that elevated power can increase self-esteem [3]. Nevertheless, research regarding the ability of power to effectively predict self-esteem has been far from conclusive. The current study drew on the theory about two different orientations (self-oriented and social-responsibility) associated with power [4] to investigate whether power predicts self-esteem through two distinct pathways: authenticity and relationship satisfaction.

### Relationship between power and self-esteem

Power is typically defined as an individual’s relative capacity to modify others’ states, by providing or withholding resources or administering punishments [2]. The current study focused on personal sense of power referring to a perception of one’s capacity to influence others, rather than control over resources [1]. The relationship between power and self–esteem was originally postulated by Kipnis (1972) who reasoned that power holders are able to effectively influence others that then leads them to believe that their capacities are superior to other people. In fact, the association between power and self–esteem is probably reciprocal. On one hand, heightened self–esteem may pave the way to power. On the other hand, variations in power may exert influence on self–esteem that is the empirical focus of the current studies. Previous evidence shows that heightened power can increase self–esteem and lowered power leads to decreases in self–esteem [3]. However, it remains unclear how power relates to self-esteem.

In definition, self-esteem refers to an evaluative response or attitude toward the self [5]. According to the *sociometer hypothesis* [6], more than an evaluation of one’s personal attribute, self-esteem is also related to how people think they are being perceived and evaluated by others. Similarly, high power contributes to both self-interested behavior (e.g., behave more consistently with one’s internal traits, Keltner et al., 2003) and other oriented behavior (perspective taking and interpersonal sensitivity, Schmid et al., 2009). Theoretically, the realization of one’s own desires would contribute to individual’s positive self-regard. On the other hand, individuals’ self-esteem might benefit from higher relationship satisfaction due to more others’ acceptance. Hence, we hypothesized that power might lead to high self-esteem through perceived positive personal and interpersonal consequences: authenticity and relationship satisfaction, respectively.

### Authenticity as a mediator between power and self-esteem

Recent research has shown that possessing power can increase individuals’ authenticity, which refers to the degree to which individuals connect with and enact their true desires in various situations [7–9]. Specifically, power will lead people to behave more consistently with their true desires [2,10], allowing them to express their true orientations and dispositions [4,11].

For many years, scholars have argued that authenticity is an important aspect of healthy psychological functioning [12]. Kernis (2003) proposes that experiencing oneself as authentic provides the basis for experiencing optimal self-esteem. Meanwhile, considerable research indicates that authenticity is particularly important in promoting self-esteem and life satisfaction [13–16]. Other research and theory proposes that power also contributes to high self-esteem. A meta-analysis revealed a positive correlation between emerging as a leader and self-esteem [17]. Furthermore, it has been found that elevated power elicited by role task can increase self-esteem [3].

In summary, power can increase feelings of authenticity, which is important to self-esteem. Other research and theory has connected power to self-esteem. We link these disparate findings into a causal path from power to authenticity to self-esteem.

### Relationship satisfaction as a mediator between power and self-esteem

Besides authenticity, a previous study showed that power can also increase interpersonal relationship satisfaction, which refers to personal satisfaction with the quality of one’s relationship with general or specific others [18]. For example, possessing high power in romantic relationships (e.g., possessing more resources or decision-making authority) is correlated with greater relationship satisfaction [19]. Meanwhile, high-power-priming individuals experience more positive emotions in interpersonal interactions, and in turn have more interpersonal sensitivity [19,20].

Furthermore, relationship satisfaction is crucial for individuals’ self-esteem. The *sociometer theory* argues that self-esteem is not merely a private self-evaluation, but also reflects the quality of one’s relationships with others [6,21]. For example, social exclusion associated with low relationship satisfaction can cause decrease in self-esteem [22]. For East Asians, who consider interpersonal relatedness to be an important aspect of their well-being, low relationship satisfaction is particularly detrimental to their self-esteem [23,24]. Given that possessing power can increase individuals’ satisfaction with interpersonal relationships, which is correlated with self-esteem, a personal sense of power might enhance self-esteem by increasing individuals’ relationship satisfaction

### Relationship between authenticity, relationship satisfaction, power, and self-esteem

In the evolutionary perspective, why humans seek power is because power is inherently related to social success and relationship with others [25,26] that are important aspects of individuals’ self-esteem. Thus, we proposed that power can enhance self-esteem through two positive results: authenticity and relationship satisfaction. The distinction between the two pathways of directing power to self-esteem is consistent with the proposition that people may associate power with both self-oriented and social responsibility goals [4,27]. Chen et al. (2001) proposed that self-oriented goals associated with power elicit behavior focused on promoting one’s own needs and interests, while social responsibility goals elicit behavior focused on responding to others’ views and needs. As positive results of wielding power in two different manners, authenticity occurs when individuals satisfy their true desires, and satisfied relationships are obtained through acceptance by others due to highly socially responsible behaviors. In turn, both increased authenticity and relationship satisfaction can enhance individuals’ self-esteem. As such, power might increase self-esteem through two distinct pathways: authenticity and relationship satisfaction.

A previous study using participants from Western cultures showed that power enhances an individual’s well-being or self-esteem through authenticity [3,9]. However, whether possessing power and authenticity leads to positive psychological functioning in East Asian cultures (e.g., China) has yet to be tested. It has been proposed that East Asian and Western cultures vary considerably in the relative emphasis on independence as opposed to interdependence [28]. Considerable evidence shows that Asians are interpersonally attuned as compared to Euro-Americans [29–31]. Because Euro-Americans are independent, their self-evaluation depends relatively more on inner authentic feelings. However, Asians’ self-evaluation is based to a greater extent on relationships with others. Here, we sought to determine whether authenticity will contribute to Chinese self-esteem, as shown in Western cultures (Kifer et al., 2013). We will further extend Kifer et al.’s (2013) study to determine whether interpersonal relationships also mediate the effects of power on self-esteem in Chinese participants.

### Overview of the Current study

The current study examined the relationships between personal sense of power, authenticity, and relationship satisfaction in the prediction of self-esteem among Chinese participants. This study aimed to answer two main questions. First, would power, authenticity, and relationship satisfaction offer unique contributions to self-esteem in Chinese participants? Second, would authenticity and relationship satisfaction both mediate the effects of power on self-esteem? To answer these questions, we investigated the relationships between power, authenticity, relationship satisfaction, and self-esteem in three related studies. Specifically, the study 1 firstly aimed to examine the relationship between personal power, authenticity, and relationship satisfaction in the prediction of self-esteem. Second, considering our participants were all Chinese who were likely to keep a high sense of communal orientation to maintain good relationships with others, the study 2 was to designed to examine whether authenticity and relationship satisfaction mediated the effects of power on self-esteem when controlling for participants’ communal orientation. At last, considering both self-esteem, power, authenticity, and relationship satisfaction were related to positive social value, the study 3 was designed to examine whether the results of Studies 1 and 2 can be replicated when controlling for participants’ level of social desirability.

## Study 1

### Methods

#### Participants and procedure

The sample comprised 104 college students (46 men, 58 women) who were recruited at a Chinese University. Ages ranged from 18 years to 33 years (*M*_*age*_ = 21.27, *SD* = 2.82). The study was approved by the Institutional Review Board of Beijing Normal University. Written informed consent was obtained from all participants, who were instructed to complete a set of paper-and-pencil questionnaires in the classroom.

#### Measures

All questionnaires were presented in their Chinese versions. The English scales were translated into Chinese, according to the standard guidelines, by a native Chinese speaker with English as a second language [32]. To control for order effects, we randomized the order of the items within each survey. All ratings were made using 5-point Likert scales, ranging from 1 (*strongly disagree*) to 5 (*strongly agree*).

#### Personal power

Dispositional power was assessed via the Sense of Power Scale [1]. This 8-item scale asks respondents to report their beliefs about the power they have. A sample item is ‘I think I have a great deal of power’ (Cronbach’s alpha = .71).

#### Self-esteem

The 10 items of the Rosenberg Self-Esteem Scale was used to assess participant’s overall evaluation of his or her worthiness as a human being [33]. A sample item is “On the whole, I am satisfied with myself” (Cronbach’s alpha = .81).

#### Authenticity

A 12-item scale developed by Wood et al. (2008) was used to assess the three facets of authenticity. The three subscales consist of: self-alienation (4 items, e.g. ‘‘I don’t know how I feel inside”), accepting external influences (4 items, e.g. ‘‘I usually do what other people tell me to do”), and authentic-living (4 items, e.g. ‘‘I always stand by what I believe in”). The Cronbach’s alpha for total authenticity was .81.

#### Relationship satisfaction

Relationship satisfaction was measured using Burns’ (1993) 7-item Relationship Satisfaction Scale in various areas of the general, not specific, relationship, including communication and openness, conflict resolution, degree of caring and affection, intimacy and closeness, satisfaction with roles in relationship, and overall relationship satisfaction. Respondents indicated their degree of relationship satisfaction with others in general. A sample item is “Overall, I am satisfied with my relationship” (Cronbach’s alpha = .82).

### Results

#### Descriptive statistics and relationship between variables

A summary of the descriptive statistics and a correlation matrix for the study variables is provided in Table 1. An inspection of the correlations revealed that power, authenticity, and relationship satisfaction were all positively related to self-esteem. Meanwhile, gender had no effects on individuals’ self-esteem, power, authenticity, and relationship satisfaction. Age was weakly correlated with only power (*r* = .20, *p* = .04), and was not correlated with self-esteem, authenticity, or relationship satisfaction. Hence, both gender and age were not included in later analyses.

**Table 1:** Descriptive statistics and bivariate correlations (Study 1).

Variable	M	SD	1	2	3	4
**1. Power**	27.71	3.78	1			
**2. Authenticity**	42.49	6.18	.44***	1		
**3. Self-esteem**	38.34	5.08	.61***	.61***	1	
**4. Relationship satisfaction**	26.09	3.66	.37***	.37***	.48***	1

*Note*. *n* = 104

***p < .001.

Considering the high correlation among self-esteem, authenticity, and power, we performed a confirmatory factor analysis (CFA) which evaluated a one-factor solution among the three constructs. The result showed one factor model did not provide adequate fit to the data (*χ* ^2^ = 418.195, *df* = 189, CFI = .642, TLI = .602, RMSEA = .109) that revealed self-esteem, power, and authenticity might not reflect the same underlying factor.

#### Authenticity and relationship mediate the effect of power on self-esteem

**Fig 1:**
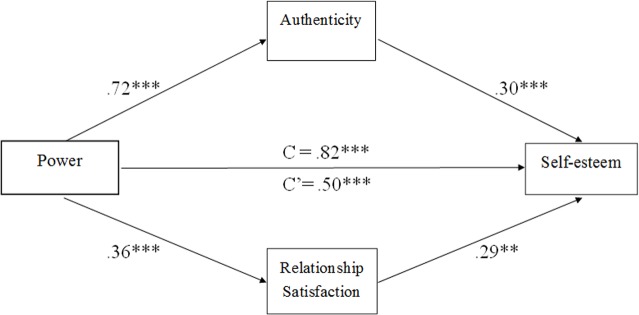
A mutiple mediation model of the association between power and self-esteem via authenticity and relationship satisfaction (*n* = 104). Note: Path estimates are standardized. **p < .01, ***p < .001.

We used an SPSS macro designed for assessing multiple mediation models [34] to examine whether authenticity and relationship satisfaction mediated the effects of power on self-esteem. Results showed that authenticity was a significant mediator, such that authenticity was positively related to power (*B* = .72), which, in turn, was positively related to self-esteem (*B* = .30). Additionally, relationship satisfaction was positively related to both power (*B* = .36) and self-esteem (*B* = .29; see Fig 1 and Table 2). Considering the reciprocal relationship between power and self-esteem, we further performed a multiple mediation analysis to examine whether authenticity and relationship satisfaction would mediate the effects of self-esteem on personal sense of power. The result shows that authenticity and relationship satisfaction might not mediate the effects of self-esteem on personal sense of power (BCa 95% CI: -.05 - .20).

**Table 2:** Indirect effects of power on self-esteem (Study 1).

Mediators	Parameter estimate	SE	BCa 95%CI
Total	.32*	.07	.20 - .50
Authenticity	.22*	.06	.11 - .37
Relationship satisfaction	.10*	.05	.03 - .23

*Note*: All bootstrapping procedures were based on 5,000 random samples with replacement. The bias-corrected and accelerated (BCa) confidence intervals (CIs) include correction for median bias and skew. CIs excluding zero are interpreted as being significant.

*p < .05.

Although the result of Study 1 was consistent with our hypothesized model, it is possible that the interrelations of the constructs could also be described by different models. One possibility is that authenticity and relationship satisfaction played different roles in the prediction of individuals’ power to their self-esteem. Specifically, authenticity might mediate the effect of power on the self-esteem only when participants’ relationship satisfaction is higher. Then, relationship satisfaction might mediate the effect of power on the self-esteem only when participants’ authenticity is higher. To exclude the possibility that authenticity (or relationship satisfaction) moderated the mediation effect of relationship satisfaction (or authenticity) between power and self-esteem, we tested two alternative models using the PROCESS macro designed for assessing multiple models [34]. *Alternative A*: We tested a plausible model that authenticity moderated the mediation effect of relationship satisfaction between power and self-esteem. The result showed that the conditional indirect effect of power on self-esteem through relationship satisfaction was not significant (power x authenticity: *t* (104) = -.24, *p* = .81). *Alternative B*: We tested a plausible model that relationship satisfaction moderated the mediation effect of authenticity in the prediction of power to self-esteem. The result showed that the conditional indirect effect of power on self-esteem through authenticity was also not significant (power x relationship satisfaction: *t* (104) = .37, *p* = .71). Thus, two alternative models that suggested authenticity (or relationship satisfaction) played a moderation role in the relationship between power and self-esteem were rejected.

The results of Study 1 showed that power, authenticity, and relationship satisfaction contributed to self-esteem independently in Chinese college students. More importantly, both authenticity and relationship satisfaction mediated the effects of power on self-esteem.

## Study 2

Chen et al. (2001) proposed that individuals with high communal orientation associate power with social responsibility goals. Meanwhile, Chinese participants are likely to keep a high sense of communal orientation to maintain good relationships with others. Therefore, communal orientation might confound the effects of relationship satisfaction in predicting self-esteem from power. The first aim of Study 2 was to examine whether authenticity and relationship satisfaction mediate the effects of power on self-esteem when controlling for participants’ communal orientation. The second aim of Study 2 was to examine whether the results of Study 1 can be generalized to adults.

### Methods

#### Participants and procedure

We recruited 191 adult Chinese participants from a professional website offering paid online tasks (male = 95, *M*_*age*_ = 33.26, *SD* = 6.04). The study was approved by the Institutional Review Board of Beijing Normal University. After having read the study information, participants then indicated their agreement with the study protocol and procedure by signifying their consent online.

#### Measures

All ratings were made using 5-point Likert scales, ranging from 1 (*strongly disagree*) to 5 (*strongly agree*). To control for order effects, we randomized the order of the items within each survey.

#### Communal orientation

The 14-item Communal Orientation Scale [35] was used to assess the degree to which a person values communally oriented interpersonal relationships by behaving communally. A sample item is “I expect people I know to be responsive to my needs and feelings” (Cronbach’s alpha = .67).

We used the same measures of personal power (Cronbach’s alpha = .73), self-esteem (Cronbach’s alpha = .78), authenticity (Cronbach’s alpha = .80), and relationship satisfaction (Cronbach’s alpha = .83) as those used in Study 1.

### Results

The descriptive statistics and bivariate correlations for all variables are presented in Table 3. As study 1 showed, power, authenticity, and relationship satisfaction were all positively related to self-esteem. The communal orientation was positively correlated with relationship satisfaction, but had no association with power, authenticity, or self-esteem. Meanwhile, both gender and age had no effects on individuals’ self-esteem, power, authenticity, and relationship satisfaction like study 1, hence we did not include age and gender in later analysis.

**Table 3:** Descriptive statistics and bivariate correlations (Study 2).

Variable	M	SD	1	2	3	4
**1. Power**	29.63	3.60	1			
**2. Authenticity**	43.85	5.28	.51***	1		
**3. Self-esteem**	34.82	4.31	.65***	.61***	1	
**4. Relationship satisfaction**	27.59	3.08	.63***	.38***	.60***	1
**5. Communal orientation**	51.21	5.00	.09	-.07	.06	.37***

*Note*. *n* = 191

***p < .001.

As study 1, we also performed a CFA which evaluated a one-factor solution among the constructs of self-esteem, power, and authenticity. The result showed that one factor model did not provide adequate fit to the data (*χ* ^2^ = 593.150, *df* = 189, CFI = .678, TLI = .643, RMSEA = .106) that showed that self-esteem, power, and authenticity might not reflect the same underlying factor.

**Fig 2:**
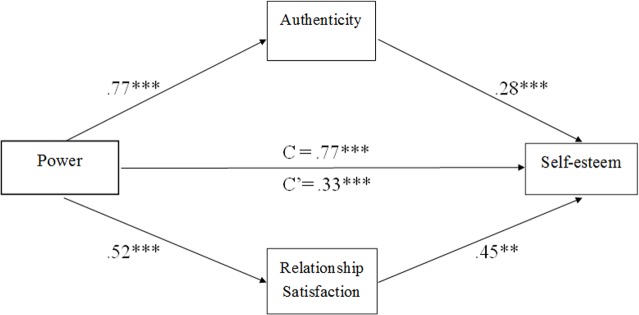
A mutiple mediation model of the association between power and self-esteem via authenticity and relationship satisfaction when controlling of communal orientation (*n* = 191). Note: Path estimates are unstandardized. **p < .01, ***p < .001.

Finally, we used an SPSS macro designed for assessing multiple mediation models [34] to examine whether authenticity and relationship satisfaction mediate the effects of power on self-esteem when controlling for participants’ communal orientation. The results showed that authenticity was a significant mediator, such that power was positively related to authenticity (*B* = .77), which, in turn, was positively related to self-esteem (*B* = .28) (see Fig 2). Additionally, relationship satisfaction was positively related to both power (*B* = .52) and self-esteem (*B* = .45). These results indicated that the association between power and self-esteem could be explained by both authenticity and relationship satisfaction, even when controlling for communal orientation (see Table 4).

**Table 4:** Indirect effects of power on self-esteem, controlling of communal orientation (Study 2).

Mediators	Parameter estimate	SE	BCa 95%CI
Total	.45*	.07	.32 - .60
Authenticity	.22*	.05	.14 - .32
Relationship satisfaction	.23*	.06	.13 - .36

*Note*: All bootstrapping procedures were based on 5,000 random samples with replacement. The bias-corrected and accelerated (BCa) confidence intervals (CIs) include correction for median bias and skew. CIs excluding zero are interpreted as being significant.

*p < .05.

Furthermore, the two alternative models were also examined as Study 1. The results showed that the conditional indirect effect of power on self-esteem through relationship satisfaction was not significant (power x authenticity: *t* (191) = .37, *p* = .71), meanwhile the conditional indirect effect of power on self-esteem through authenticity was not significant (power x relationship satisfaction: *t* (191) = -1.25, *p* = .21) when controlling for participants’ communal orientation. Hence, two alternative models that suggested authenticity (or relationship satisfaction) played a moderation role in the relationship between power and self-esteem were rejected.

## Study 3

To determine whether the multiple mediation effects of authenticity and relationship satisfaction in predicting power from self-esteem can be explained by participants’ social desirability responses, the aim of Study 3 was to examine whether the results of Studies 1 and 2 can be replicated when controlling for participants’ level of social desirability.

### Methods

#### Participants and procedure

We recruited 210 adult Chinese participants from a professional website offering paid online tasks (*M*_*age*_ = 31.90, *SD* = 7.43). Participants varied considerably in profession (for example, 7.1% students, 16% administrative staffs, and 22% managerial personnel), socioeconomic status (from RMB 1000 to 20,000 monthly income), education (ranging from 9.1% high school degree to 13% master’s degree), and professional seniority (50% elementary and 42% senior). The study was approved by the Institutional Review Board of Beijing Normal University. After having read the study information, participants indicated their agreement with the study protocol and procedure by signifying their consent online.

#### Measures

All ratings were made using 5-point Likert scales, ranging from 1 (*strongly disagree*) to 5 (*strongly agree*). To control for order effects, we randomized the order of the items within each survey.

#### Social desirability

To reduce participant burden as much as possible, we used the 5-item Social Desirability Response Set-5 [36] to assess the degree to which a person behaves in a socially approved manner. A sample item is “I am always courteous even to people who are disagreeable” (Cronbach’s alpha = .78).

We used the same measures of personal power (Cronbach’s alpha = .88), self-esteem (Cronbach’s alpha = .90), authenticity (Cronbach’s alpha = .93), and relationship satisfaction (Cronbach’s alpha = .93) as those used in Study 1.

### Results

The descriptive statistics and bivariate correlations for all variables are presented in Table 5. As predicted, power, authenticity, and relationship satisfaction were all positively related to both self-esteem and social desirability. Meanwhile, age had no effects on individuals’ self-esteem, power, authenticity, and relationship satisfaction like study 1, hence we did not include age in later analysis.

**Table 5:** Descriptive statistics and bivariate correlations (Study 3).

Variable	M	SD	1	2	3	4
**1. Power**	29.37	5.37	1			
**2. Authenticity**	38.38	6.88	.65***	1		
**3. Self-esteem**	39.12	6.95	.76***	.68***	1	
**4. Relationship satisfaction**	27.70	4.52	.75***	.48***	.72***	1
**5. Social desirability**	.86	1.37	.46***	.46***	.52***	.55***

*Note*. *n* = 210

***p < .001.

As study 1 and 2, we performed a CFA which evaluated a one-factor solution among the constructs of self-esteem, power, and authenticity. The result showed that one factor model did not provide adequate fit to the data (*χ* ^2^ = 1084.003, *df* = 189, CFI = .705, TLI = .672, RMSEA = .151) that showed that self-esteem, power, and authenticity might not reflect the same underlying factor.

**Fig 3:**
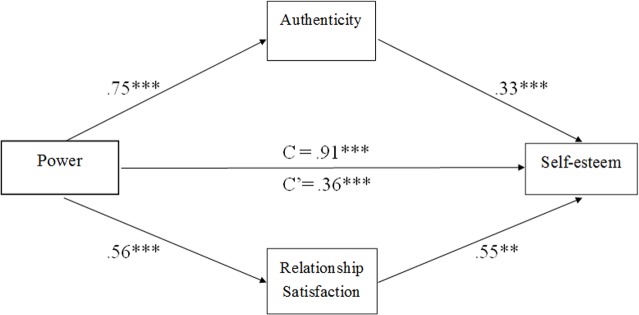
A mutiple mediation model of the association between power and self-esteem via authenticity and relationship satisfaction when controlling of social desirability (*n* = 210). Note: Path estimates are unstandardized. **p < .01, ***p < .001.

Then, we used an SPSS macro designed for assessing multiple mediation models [34] to examine whether authenticity and relationship satisfaction mediate the effects of power on self-esteem when controlling for participants’ social desirability. The results showed that authenticity was a significant mediator, such that power was positively related to authenticity (*B* = .75), which, in turn, was positively related to self-esteem (*B* = .33) (see Fig 3). Additionally, relationship satisfaction was positively related to both power (*B* = .56) and self-esteem (*B* = .55). These results indicated that the association between power and self-esteem could be explained by both authenticity and relationship satisfaction, even when controlling for social desirability (see Table 6).

**Table 6:** Indirect effects of power on self-esteem, controlling of social desirability (Study 3).

Mediators	Parameter estimate	SE	BCa 95%CI
Total	.56***	.05	.42 - .69
Authenticity	.33***	.05	.16 - .36
Relationship satisfaction	.55***	.07	.18 - .46

*Note*: All bootstrapping procedures were based on 5,000 random samples with replacement. The bias-corrected and accelerated (BCa) confidence intervals (CIs) include correction for median bias and skew. CIs excluding zero are interpreted as being significant.

***p < .05.

Furthermore, the two alternative models were also examined as Study 1 and 2. The results showed that the conditional indirect effect of power on self-esteem through relationship satisfaction was not significant (power x authenticity: *t* (210) = -.40, *p* = .69), meanwhile the conditional indirect effect of power on self-esteem through authenticity was not significant (power x relationship satisfaction: *t* (210) = -1.04, *p* = .30) when controlling for participants’ social desirability. Hence, two alternative models that suggested authenticity (or relationship satisfaction) played a moderation role in the relationship between power and self-esteem were rejected.

## Discussion

The present study investigated whether authenticity and relationship satisfaction mediate the effects of power on self-esteem in Chinese participants. The results of the three studies are consistent with our hypothesis that power, authenticity, and relationship satisfaction each provide a unique contribution in predicting self-esteem. More importantly, multiple mediation analyses revealed that both authenticity and relationship satisfaction mediate the effects of power on self-esteem, even when controlling for participants’ communal orientation and social desirability. This finding offers the first evidence for the mediating role of authenticity and relationship satisfaction in the ability of power to predict self-esteem in a sample of Chinese people. This finding further contributes to understanding how power effectively predicts self-esteem.

In demonstrating the proposed associations, our results contribute to the literature in two important ways. First, we have replicated much of the work of Kifer et al. (2013), who examined conceptually similar constructs describing how power leads to improved life satisfaction through the pathway of authenticity. The unique contribution of authenticity and relationship satisfaction to Chinese self-esteem reveals that Chinese individuals do not conceal true desires or feelings to maintain harmonious relationship with others and then enhance positive self-regard.

We also extended Kifer et al.’s (2013) findings by revealing relationship satisfaction as another independent mediator of the ability of power to predict self-esteem. Traditionally, conceptual and operational definitions of power have focused on control over others [1] as encouraging people to behave more authentically [10,11] and enhancing their well-being [9]. Otherwise, power is a social-relational concept, and an individual’s power can only be understood in relation with other individuals [1]. This is particularly true for East Asians, who consider interpersonal relatedness to be very important in their daily lives [23,24]. For this reason, relationship satisfaction, as well as authenticity, was found to mediate the relationship between power and self-esteem in Chinese participants.

Several aspects of the present research warrant further investigation. First, our measures focused primarily on self-reported personal power, as opposed to positional power or need for power. Future research should test the generalizability of our findings by using more objective measures of power, such as socioeconomic status, hierarchies, or contextual power [4], and test whether people’s desire for power is composed of desires for self-authenticity and relationship satisfaction. Furthermore, the self-reported data might be a main reason that leads to the high correlation between self-esteem and power. To reveal the true relationship between self-esteem and power, future study was suggested to use more objective indices to measure individuals’ power and self-esteem, such as other’s evaluation or implicit self-esteem. The second limitation is the cross-sectional design make it impossible to draw causal conclusions. Future longitudinal studies will contribute to promoting a comprehensive understanding of the relationships among power, self-esteem, authenticity, and relationship satisfaction. Third, considering all our participants are Chinese and the lack of cross-cultural data, it is needed for future research to test for cross-cultural differences in the effects of power on authenticity/relationship satisfaction and self-esteem.

In conclusion, the current study offers an integrated model to interpret how various components of self-esteem co-vary within individuals. Such a model advances our understanding of how power predicts self-esteem. We expect that this study and the proposed model will provide a foundation that future research can build upon in order to address these and other issues regarding power and self-esteem.

## Supporting Information

S1 Dataset (SAV)

S2 Dataset (SAV)

S3 Dataset (SAV)
